# Plasma Neurodegeneration and AD Biomarkers Identify Populations With Rapid Cognitive Decline

**DOI:** 10.1093/geroni/igaf122.211

**Published:** 2025-12-31

**Authors:** Josef Coresh, James Pike, Nicholas Reed, Priya Palta, Jennifer Deal, Kevin Sullivan, Bharat Thyagarajan, Frank Lin

**Affiliations:** New York University, New York, New York, United States; New York University, New York, New York, United States; New York University Grossman School of Medicine, New York, New York, United States; University of North Carolina School of Medicine, Chapel Hill, North Carolina, United States; Johns Hopkins Bloomberg School of Public Health, Baltimore, Maryland, United States; University of Mississippi Medical Center - The MIND Center, Jackson, Mississippi, United States; University of Minnesota, Minneapolis, Minnesota, United States; Johns Hopkins Bloomberg School of Public Health, Baltimore, Maryland, United States

## Abstract

Predicting rapid cognitive decline is valuable for patient counseling and targeting clinical trials to slow down this rate of decline. We used neurodegeneration and AD biomarkers measured in the ARIC cohort (amyloid-β 42 to amyloid-β 40 ratio, p-tau 181, NFL, and GFAP on frozen plasma collected in 2011-13 using the Quanterix SiMoA platform, N = 1825) to develop a risk score for the rate of decline in global cognition factor score and validated its performance among participants of the ACHIEVE clinical trial (same 4 biomarkers assayed using the Alamar CNS protein panel, N = 502 at the 3-year follow-up). Biomarkers were standardized within platforms using inverse-normal transformation. Cognitive decline risk scores developed in ARIC were applied to ACHIEVE for validation. Biomarker risk scores developed in ARIC validated in ACHIEVE with the upper quintile having a mean (95% CI) progression rate of -0.123 (-0.137, -0.108) in ARIC, -0.111 (-0.158, -0.065) in ACHIEVE (compared to mean of -0.012 in the total ACHIEVE validation sample). Adding age and Mini Mental State Exam did not improve the risk scores. The power of a clinical trial would change from 5% to 96% as mean annualized cognitive decline increases from -0.012 to -0.111 (selected top risk quintile; a progression SD of 0.25) in a trial of 800 participants and a 20% reduction in cognitive decline from an intervention. In conclusion, risk scores based on plasma biomarkers allow for identification of subgroups likely to experience rapid cognitive decline and may increase the power of cognitive decline clinical trials.

